# Role of Python-Based Statistical Analysis in Comparing Polypropylene and Polyester Mesh Implants in Lichtenstein Hernia Repair: A Retrospective Comparative Cohort Study

**DOI:** 10.7759/cureus.93415

**Published:** 2025-09-28

**Authors:** Sameh M Salem, Andrey Vitalevitch Protasov, Mekhaeel Shehata Fakhry Mekhaeel

**Affiliations:** 1 Operative Surgery and Clinical Anatomy, Peoples’ Friendship University of Russia (RUDN University), Moscow, RUS

**Keywords:** ai and machine learning, artificial intelligence, femoral hernia, hernia, inguinal hernia repair, lichtenstein, polyester mesh, polypropylene mesh, python program, surgical mesh

## Abstract

Introduction: The choice of mesh material in Lichtenstein hernia repair remains a topic of discussion. This study aimed to compare short-term outcomes of polypropylene versus polyester mesh using statistical analysis in Python (Python Software Foundation).

Methods: A single-center, retrospective cohort study was conducted on 58 patients undergoing Lichtenstein repair. Patients were allocated to polypropylene (n=38) or polyester (n=20) mesh groups based on the surgeon's preference and material availability. Primary outcomes were complication rates and operative time. Statistical analysis was performed using Python (version 3.8.0) with SciPy and Pandas libraries.

Results: Significant baseline differences were noted; the polypropylene group was older (58.1 vs. 50.1 years, p=0.024) and had larger hernia volumes (138.5 vs. 53.9 cm³, p=0.023). Despite this, no significant differences were found in operative time (52.0 vs. 48.5 minutes, p=0.622), hospital stay (5.0 vs. 4.8 days, p=0.550), or complication rates (7.9% vs. 5.0%, p=1.000) between the polypropylene and polyester groups, respectively.

Conclusions: In this retrospective analysis, we observed no significant short-term differences in outcomes between mesh types. However, these findings are limited by the study's retrospective design, small sample size, and baseline imbalances. The choice of mesh may continue to be based on the surgeon's experience, but larger randomized trials are needed for definitive conclusions.

## Introduction

The global burden of groin hernias remains significant, underscoring the continued importance of refining surgical repair techniques [1,2]. Inguinal hernias represent a common surgical pathology, with well-documented risk factors including male gender, advanced age, and increased intra-abdominal pressure [3,4]. The Lichtenstein tension-free repair, a gold standard open approach, is a prevalent and cost-effective procedure for managing this condition [5,6].

The choice of mesh material is a critical factor influencing postoperative outcomes. Polypropylene mesh, a common choice, is non-absorbable, has high tensile strength, and is hydrophobic. Polyester mesh, also widely used, is multifilamentous, hydrophilic, and may be coated to inhibit adhesions [7,8]. A rigorous, modern comparison of their performance is a relevant clinical question.

The accurate statistical analysis of surgical outcomes is paramount. In this context, programming languages like Python (Python Software Foundation) offer superior advantages over traditional tools, including scalable analysis of complex datasets, a rich ecosystem of scientific libraries, and the robust implementation of advanced statistical models [9-11].

Therefore, the aim of this study was to compare outcomes of polypropylene versus polyester mesh in Lichtenstein repair using statistical analysis in Python.

## Materials and methods

Study design and ethical considerations

This was a single-center, retrospective comparative cohort study conducted at the clinical base of the Department of Operative Surgery and Clinical Anatomy at Peoples’ Friendship University of Russia (RUDN University), Moscow, Russian Federation. The study utilized anonymized historical data from patient records. The RUDN University Ethics Committee granted a formal exemption for this study, as it involved the analysis of existing, anonymized data without any prospective intervention or breach of patient privacy, in accordance with the national regulations and the Declaration of Helsinki.

Patient population and selection

We analyzed the records of 58 patients who underwent Lichtenstein hernia repair surgery within the previous three years. Patients were allocated into two groups based on the type of mesh implanted during their original surgery: (i) Group A (n=38): Hernia repaired with a polypropylene mesh; (ii) Group B (n=20): Hernia repaired with a polyester mesh.

The assignment to mesh type was non-randomized and was determined by the surgeon's preference and product availability.

Inclusion and exclusion criteria

Inclusion criteria comprised individuals aged 20-71 years with a unilateral, primary, uncomplicated inguinal hernia undergoing elective Lichtenstein repair. Exclusion criteria were emergency presentations, age <20 years or >71 years, bilateral or recurrent hernias, complex incarcerated hernias, and cases performed laparoscopically.

Data collection

The following data were extracted from patient medical records: (i) Preoperative data: Sex, age, duration of symptoms, hernia type and location, hernia defect size, and associated comorbidities; (ii) Operative data: Type of anesthesia, total operative time (minutes), and size of mesh implanted (cm²); (iii) Postoperative data: In-hospital complications (e.g., hematoma, seroma, surgical site infection), total length of hospital stay (days), and outcomes from short-term follow-up visits.

Study outcomes

The primary outcomes were the incidence of postoperative complications and recurrence rates. Secondary outcomes included operative time and hospital length of stay. Patients were followed up clinically at two weeks, one month, three months, and 12 months postoperatively.

Surgical technique and materials

All procedures were performed by experienced surgeons using the standard Lichtenstein tension-free repair technique. For Group A, a lightweight polypropylene mesh (B. Braun, Germany) was used. For Group B, a knitted polyester mesh (Medtronic, USA) was used.

Statistical analysis

All statistical analyses were performed using the Python programming language (version 3.8.0) with the SciPy and Pandas libraries [10,11]. Descriptive statistics were presented as arithmetic mean with standard deviation (SD) for normally distributed continuous variables, median with interquartile range (IQR) for non-normal data, and frequencies (n, %) for categorical variables. For comparative analysis, the Student’s t-test or Mann-Whitney U test was used for continuous variables, and the Chi-square or Fisher’s exact test was used for categorical variables, as appropriate. A p-value of <0.05 was considered statistically significant.

## Results

A retrospective analysis was conducted on a cohort of 58 patients who underwent elective Lichtenstein tension-free hernia repair, stratified into two groups based on the implanted mesh material. The polypropylene mesh group (Group A) comprised 38 patients, while the polyester mesh group (Group B) included 20 patients. An initial comparative assessment of preoperative demographics and clinical characteristics revealed clinically relevant and statistically significant disparities between the groups, underscoring the non-randomized nature of the study.

As detailed in Table 1, the patient population in Group A was, on average, eight years older than those in Group B (58.1 ± 10.1 years vs. 50.1 ± 12.5 years, p=0.024). More notably, a striking difference was observed in the complexity of the presenting hernias. The mean hernia volume in the polypropylene group was over two-and-a-half times larger than that in the polyester group (138.5 ± 259.3 cm³ vs. 53.9 ± 87.1 cm³, p=0.023). This combination of advanced age and larger hernia defects positioned Group A as a higher-risk cohort at baseline. Other characteristics, including sex distribution, symptom duration, hernia type (direct vs. indirect), and comorbidity burden, were well-balanced, suggesting that the surgeon's preference or material availability may have been influenced by perceived hernia complexity.

**Table 1:** Baseline and operative characteristics of the study cohort Data presented as mean ± SD or n (%). P-values are calculated using Welch's t-test for continuous variables and Fisher's exact test for categorical variables. *P-value <0.05, statistically significant SD: Standard deviation

Characteristic	Group A (Polypropylene), n=38	Group B (Polyester), n=20	p-value
Demographics			
Age, years (mean ± SD)	58.1 ± 10.1	50.1 ± 12.5	0.024*
Sex male, n (%)	34 (89.5%)	20 (100%)	0.297
Hernia characteristics			
Duration, months (mean ± SD)	35.0 ± 39.1	22.8 ± 25.2	0.143
Volume, cm³ (mean ± SD)	138.5 ± 259.3	53.9 ± 87.1	0.023*
Type (direct), n (%)	19 (50%)	8 (40%)	0.584
Comorbidities, n (%)	18 (47.4%)	9 (45.0%)	1.000

The primary surgical and postoperative outcomes are presented in Table 2. Despite the baseline imbalances suggesting greater anatomical complexity in Group A, the intraoperative and short-term recovery metrics were remarkably similar between the two groups. The mean operative time for the polypropylene group was 52.0 ± 17.7 minutes, compared to 48.5 ± 18.9 minutes for the polyester group. This difference of 3.5 minutes was not statistically significant (95% confidence interval (CI) (-10.4, +17.4), p=0.622), indicating that the repair of larger hernias with polypropylene mesh did not confer a substantial time penalty.

**Table 2:** Comparison of operative and postoperative outcomes between mesh groups Data presented as mean ± SD or n (%). The mean difference (with 95% CI) is reported for continuous variables; OR (with 95% CI) is reported for the composite outcome of postoperative complications. P-values are calculated using Welch's t-test for continuous variables and Fisher's exact test for categorical variables. ¹Fisher's exact test OR: Odds ratio; SD: Standard deviation; CI: Confidence interval

Outcome	Group A (Polypropylene)	Group B (Polyester)	Mean Difference / Effect (95% CI)	p-value
Operative time, min	52.0 ± 17.7	48.5 ± 18.9	+3.5 (-10.4 to +17.4)	0.622
Postoperative complications, n (%)	3 (7.9%)	1 (5.0%)	OR: 1.63 (0.16 to 17.0)	1.000¹
Postoperative pain	3 (7.9%)	1 (5.0%)	-	-
Hospital stay, days	5.0 ± 1.2	4.8 ± 1.0	+0.2 (-0.4 to +0.8)	0.550

This equivalence extended into the postoperative period. The length of hospital stay was nearly identical, with a mean of 5.0 ± 1.2 days for Group A and 4.8 ± 1.0 days for Group B (mean difference +0.2 days, 95% CI (-0.4, +0.8), p=0.550). The overall incidence of short-term complications was low and comparable between groups. In the polypropylene group, three patients (7.9%) experienced complications, all of which were transient postoperative pain manageable with oral analgesics. Similarly, one patient (5.0%) in the polyester group reported postoperative pain. The odds ratio (OR) for complications was 1.63, but with a wide 95% CI (0.16 to 17.0) that crossed unity, confirming the lack of a statistically significant association (p=1.000). Critically, no other complications such as surgical site infections, seromas, hematomas, or early recurrences were recorded in either group during the six-month clinical follow-up period.

In summary, while the group allocated to polypropylene mesh presented with significant preoperative risk factors, the outcomes following Lichtenstein repair were virtually indistinguishable from those in the lower-risk polyester group across all measured short-term parameters.

## Discussion

This single-center, retrospective cohort study utilized Python-based statistical analysis to compare short-term outcomes between polypropylene and polyester mesh in Lichtenstein tension-free hernia repair. The central finding of our analysis is that, within the constraints of our study design, we observed no statistically significant differences in operative time, length of hospital stays, or short-term complication rates between the two mesh types.

This absence of significant difference in key short-term outcomes aligns with several previous investigations. For instance, a randomized controlled trial by Nikkolo et al. (2010) concluded that polyester mesh could not be recommended over polypropylene based on postoperative pain or quality of life, as they found no differences in recurrence or infection rates [12]. Similarly, Ramshaw et al. (2003), in a large multicenter study on laparoscopic repair, reported no mesh-related complications and comparable outcomes between the materials [13]. Our findings contribute to this body of evidence by suggesting that, in the context of open Lichtenstein repair, both synthetic meshes may offer equivalent performance in the immediate postoperative period.

However, a critical nuance of our study is the significant baseline imbalance between the groups. The polypropylene group consisted of significantly older patients with larger hernia volumes, representing a cohort with inherently more complex surgical anatomy and potentially higher risk for complications. The fact that operative times were not significantly prolonged in this group, despite tackling more substantial hernias, could be interpreted as suggesting that the handling and placement of polypropylene mesh does not present a greater technical challenge in such scenarios. Moreover, the low and comparable complication rates, even in this higher-risk group, are reassuring and could hint at the robustness of both materials. This observation invites the hypothesis that the surgeon's familiarity and technical execution of the Lichtenstein procedure may be a more critical determinant of early success than the specific choice between these two mesh materials.

The use of Python for our statistical analysis provided a robust and reproducible framework for handling these complex comparisons. Libraries such as Pandas facilitated efficient data manipulation, while SciPy ensured the application of appropriate statistical tests, including Welch's t-test to account for unequal group sizes and variances. This approach underscores the utility of modern programming tools in clinical research for enhancing analytical rigor.

Limitations and strengths

Our study's conclusions must be interpreted within the context of its limitations. The primary limitation is its retrospective, non-randomized design, which introduces significant potential for selection and confounding biases, as clearly evidenced by the imbalanced baseline characteristics. The assignment of mesh type based on the surgeon's preference and availability, rather than random allocation, means that unmeasured factors (e.g., subtle surgeon bias towards using a specific mesh for perceived more complex cases) could have influenced the results. Second, the relatively small sample size, particularly the modest size of the polyester group (n=20), substantially limits the statistical power of the study. This increases the risk of a Type II error, meaning that we may have failed to detect clinically relevant differences that a larger trial would identify. Third, the short-term follow-up period of six months precludes any assessment of long-term outcomes, most critically chronic groin pain and hernia recurrence, which are paramount in evaluating hernia repair materials. Finally, as a single-center study, the generalizability of our findings may be limited.

Despite these limitations, the study has strengths, including the use of a standardized surgical technique by experienced surgeons and a rigorous, transparent statistical methodology using modern computational tools.

Future directions

Overall, this retrospective analysis found no evidence of superior short-term outcomes for either polypropylene or polyester mesh in Lichtenstein hernia repair. The comparable results, even in the face of baseline imbalances, suggest that both materials are viable options. However, given the significant methodological limitations, these findings are preliminary and should be considered hypothesis-generating rather than definitive. The choice of mesh may appropriately continue to rest on the surgeon's experience, cost, and availability. Ultimately, our study underscores the necessity for a well-designed, prospective, randomized controlled trial with adequate power, long-term follow-up, and stratification for known risk factors to provide a conclusive comparison of these two commonly used mesh materials.

## Conclusions

In this retrospective comparative analysis, Python-based statistical analysis found no significant short-term differences in outcomes between polypropylene and polyester mesh in Lichtenstein hernia repair. However, these results are tempered by significant baseline group imbalances, a small sample size, and the limitations inherent to the retrospective design. Therefore, they should be interpreted with caution. The choice of mesh may reasonably continue to be based on the surgeon's experience and preference. Future prospective, randomized studies with larger sample sizes and long-term follow-up are needed to definitively establish the non-inferiority of one material over the other for this common procedure.
